# MicroRNAs as Biomarkers in Thyroid Carcinoma

**DOI:** 10.1155/2017/6496570

**Published:** 2017-09-06

**Authors:** Marilena Celano, Francesca Rosignolo, Valentina Maggisano, Valeria Pecce, Michelangelo Iannone, Diego Russo, Stefania Bulotta

**Affiliations:** ^1^Department of Health Sciences, Magna Graecia University of Catanzaro, 88100 Catanzaro, Italy; ^2^Department of Internal Medicine and Medical Specialties, Sapienza University of Rome, 00161 Rome, Italy; ^3^CNR, Institute of Neurological Sciences, Section of Pharmacology, Roccelletta di Borgia, 88021 Borgia, Italy

## Abstract

Optimal management of patients with thyroid cancer requires the use of sensitive and specific biomarkers. For early diagnosis and effective follow-up, the currently available cytological and serum biomarkers, thyroglobulin and calcitonin, present severe limitations. Research on microRNA expression in thyroid tumors is providing new insights for the development of novel biomarkers that can be used to diagnose thyroid cancer and optimize its management. In this review, we will examine some of the methods commonly used to detect and quantify microRNA in biospecimens from patients with thyroid tumor, as well as the potential applications of these techniques for developing microRNA-based biomarkers for the diagnosis and prognostic evaluation of thyroid cancers.

## 1. Introduction

Thyroid cancer is the most frequently diagnosed endocrine malignancy, and its prevalence has increased markedly over the last decade [1]. Neoplastic transformation can occur in either the follicular or parafollicular cells of the gland. In the former case, the results range from differentiated tumors—papillary thyroid carcinomas (PTCs), follicular thyroid carcinomas (FTCs), and Hürthle cell carcinomas—to the rarer poorly differentiated and anaplastic thyroid carcinomas (PDTCs and ATCs, resp.). Transformation of the parafollicular cells produces medullary thyroid carcinomas (MTCs). Approximately, a percentage of MTCs are familial, and this category includes those diagnosed as part of the multiple endocrine neoplasia type 2 syndrome [2]. Approximately, 80% of all differentiated thyroid carcinomas (DTCs) are PTCs. These tumors have a very good prognosis, thanks to the available tool (cytological examination of fine-needle aspiration biopsy (FNAB)) which allows an early diagnosis and the efficacy of the current treatment. It involves surgery and radioactive iodine to eliminate residual and/or locoregionally recurrent disease and, in some cases, also distant metastases. This approach is not an option for patients with MTCs or for those whose tumors (PDTCs and ATCs for the most part) are no longer able to concentrate iodine. This defect is the result of impaired expression/function of the sodium/iodine symporter (NIS) or thyroperoxidase (TPO) caused by oncogene-activated signaling that leads to thyrocyte dedifferentiation [3–5]. For these tumors, novel therapeutic strategies are being actively investigated [6, 7].

For many years, assays of serum thyroglobulin and calcitonin levels have played important roles in the diagnosis and follow-up of thyroid cancer [8, 9]. Thyroglobulin is produced exclusively by follicular thyroid cells. In patients with DTC who have undergone total thyroidectomy and radioiodine remnant ablation, its presence in the serum is thus considered a marker of persistent or recurrent disease (locoregional or at distant site metastases) [10]. Calcitonin, a product of the parafollicular C-cells, serves a similar purpose in the follow-up of patients operated on for MTC [11]. Both markers, however, have several well-documented limitations involving specificity and sensitivity [11, 12], and with the increasing prevalence of thyroid malignancy, the need for noninvasive thyroid cancer biomarkers with higher accuracy, sensitivity, and specificity has become more pressing. Interest in this field has been sparked by our increasing understanding of the expression of microRNAs (miRNAs) in patients with thyroid tumors [13]. In this review, we will examine the growing body of evidence supporting the use of these small, noncoding RNA species to diagnose and predict the behavior of thyroid cancers, as well as the techniques currently used to detect and quantify their presence in tissues and other biological samples.

## 2. miRNAs in Thyroid Cancer

miRNAs are endogenous, noncoding RNAs with lengths ranging from 19 to 25 nucleotides. They play major roles in the posttranscriptional regulation of gene expression [14–16]. In general, miRNAs downmodulate the expression of a target gene by diminishing the stability of its transcript and/or inhibiting its translation [14–16]. By reducing the abundance of specific proteins in this manner, miRNAs exert fundamental modulatory effects on many physiological processes, including those involved in pre- and postnatal developments. Therefore, it is not surprising that their dysregulated expression is a feature of several pathological conditions, including neoplastic disease [17–20]. The first description of a link between aberrant miRNA expression and cancer was published in 2002 [21]. Since then, the number of miRNAs known to be encoded by the human genome has grown rapidly. A recent look at the miRBase database revealed over 2000 annotated human miRNAs [22], and their numbers are expected to increase [23].

The first published information on the role of miRNAs in thyroid tumorigenesis emerged in 2005 [24] and was followed by several other studies focusing on this issue. Thyroid tumors (and other cancers as well) display alterations involving various components of the machinery responsible for the complex process of miRNA biogenesis. Downregulated transcription of DICER has recently been observed in malignant thyroid tissues and cell lines, as compared with normal thyroid tissues and benign thyroid neoplasms, and this alteration was correlated with features indicative of tumor aggressiveness (extrathyroidal extension, lymph node and distant metastases, and recurrence) and with the presence of the *BRAFV600E* mutation [25]. A large cohort study of MTCs found that tumors harboring *RET* mutations exhibited upregulated expression of certain genes involved in miRNA biogenesis, as compared with their RET-wildtype counterparts, while no significant differences were observed between the expression levels of these genes in *RAS*-mutant and *RAS*-wildtype MTCs [26].

Specific patterns of miRNA expression have also been identified in a large number of studies performed on thyroid carcinomas [21, 27–30], and several miRNAs were found overexpressed or downregulated in major types of thyroid tumors [13, 31, 32]. Recent meta-analyses have attempted to provide a clearer overview of the miRNAs most commonly dysregulated in specific thyroid cancer histotypes. Several groups have reported overexpression of miRNA-146b, miRNA-221, miRNA-222, and miRNA-181b in PTCs, as compared with levels in normal thyroid tissues, and this upregulation is positively correlated with tumor aggressiveness [33–37]. Three of these four miRNAs, miRNA-146b, miRNA-221, and miRNA-222, are also upregulated in FTC, Hürthle cell thyroid carcinomas, and ATC [38–40]. In contrast, miRNA-197 and miRNA-346 are upregulated specifically in FTC [29, 41]. Members of the miRNA-17-92 cluster are highly expressed in ATC, as they are in other aggressive cancers [39], suggesting that dysregulation of this miRNA cluster influences the oncogenic process. Of note, increased expression of miRNAs-21, miRNA-183, and miRNA-375 has been associated with persistent and metastatic disease in MTC patients [42]. miRNA downregulations appear to be more variably associated with specific types of thyroid cancer. An exception is the downregulated expression of miRNAs belonging to the miRNA-200 and miRNA-30 families, which is associated exclusively with ATCs and is therefore suspected to play key roles in the acquisition of particularly aggressive tumor phenotypes [13, 39].  Table 1 shows the main miRNA dysregulations found in thyroid tumors, together with their documented associations with oncogenic mutations and their validated molecular targets. Among the latter, a functional role in oncogenic transformation of thyroid cancer cells is played by proto-oncogene receptor tyrosine kinase (KIT), C-X-C motif chemokine ligand 12 (CXCL12), connective tissue growth factor (CTGF), NF-kB, programmed cell death 4 (PDCD4), and yes-associated protein (YAP) (see Table 1), all involved in the regulation of cell proliferation, migration, invasion, and survival.

## 3. miRNA Detection in Biological Samples

miRNA expression patterns can be rich sources of biological information. Analysis of variations in these patterns can provide clues as to how different cellular processes are modulated under both physiological and pathological conditions [54]. Various diseases are associated with significant changes in the miRNA profile of involved tissues, and most of these changes have been reported in different kinds of cancer [55]. miRNAs display good stability in a variety of human biospecimens, including cell lines, fresh-frozen and formalin-fixed tissues, FNAB, blood plasma and serum, and urine [56]. Moreover, their levels provide more immediate and also more specific information on current physiological and pathological conditions than other molecules in these specimens [57]. Their importance in the modulation of gene expression and their remarkable stability in human biospecimens have led to develop a variety of approaches, and platforms have been developed to isolate and study miRNA expression, with the aim to identify profiles or single miRNAs associated with specific pathological condition.

**Figure 1:**
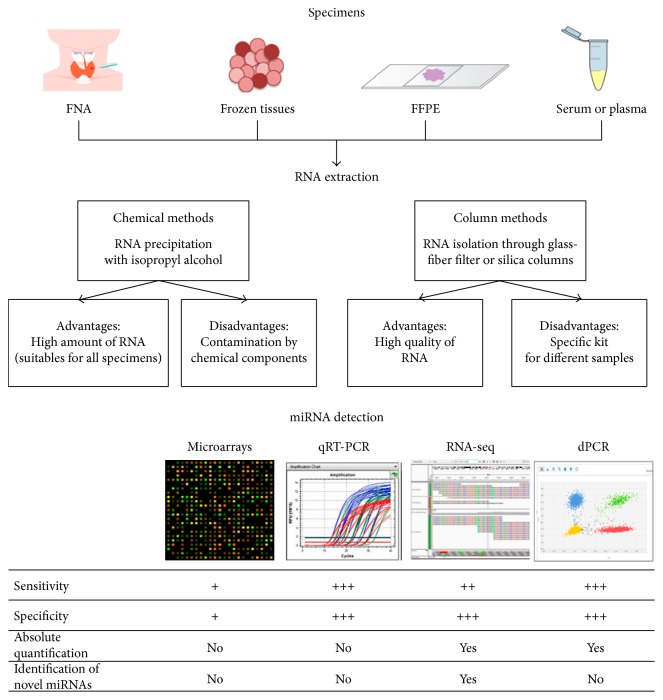
miRNA detection workflow. miRNAs can be isolated from different biospecimens. To isolate miRNAs, widely used methods are chemical and column-based techniques. After quantification step, samples are ready for miRNA profiling. Among widely used techniques, there are four established methods: microarray, quantitative PCR (qRT-PCR), massive parallel sequencing (RNA-seq), and digital PCR (dPCR). The sensitivity and specificity are classified as follows: + (low), ++ (moderate), +++ (high). FFPE: formalin-fixed paraffin embedded; FNA: fine-needle aspiration.

miRNA profiling begins with the isolation of total RNA. The extraction protocols used for this purpose are often slightly modified to enrich the fraction containing miRNAs and other small RNA species. Widely used methods for miRNA extraction fall into two main categories: chemical methods and column-based methods. The principal advantages and disadvantages of each are summarized in Figure 1.

The isolated miRNA is then quantified and subjected to quality assessment. The quantity obtained is specimen specific, whereas the quality of the RNA depends on the extraction method used. The RNA is then ready for miRNA profiling. Four well-established methods are currently used to analyze miRNA expression: microarrays, quantitative reverse-transcription PCR (qRT-PCR), high-throughput sequencing (RNA-seq), and digital PCR (dPCR).

Microarray analysis was one of the first methods used for parallel analysis of large numbers of miRNAs. The miRNAs in a biological sample are labeled using fluorescent, chemical, or enzymatic techniques and then hybridized to DNA-based probes on the array. Microarray-based profiling allows rapid processing of the high number, and its cost is relatively low cost. However, it is the least sensitive and least specific of the miRNA profiling methods, and it does not allow the identification of novel targets [58].

QRT-PCR is probably the most popular method currently used for miRNA detection. It entails reverse transcription of miRNA to cDNA, followed by real-time monitoring of the accumulation of polymerase reaction products. Commercially available, customizable plates and microfluidic cards can be designed to examine a small set of miRNAs or to provide more comprehensive coverage. For qRT-PCR detection of hundreds of miRNAs, platforms are available with preplated PCR primers distributed across microfluidic cards containing nanoliter-scale wells. This approach is more specific and sensitive than microarray profiling. An internal control must be used for relative quantification of the expression; a standard curve can be used to obtain absolute quantification. Like microarray profiling, qRT-PCR cannot identify novel miRNAs [59].

RNA-seq is currently the most expensive technique for miRNA profiling, but it is also the most informative. It provides quantification data as well as sequence data and can therefore be used to identify novel miRNAs or sequence variations. A cDNA library of small RNAs is prepared from the samples of interest. This is followed by an adaptor ligation step and immobilization of the cDNA on a support (solid phase for solid-phase PCR, bead-based for emulsion PCR). These steps are followed by massively parallel sequencing of millions of cDNA molecules from the library. This approach allows simultaneous analysis of the expression patterns of a huge number of targets [60].

Digital PCR allows quantitative analysis of miRNA expression without internal controls. It is the most sensitive technique and the only one that can directly quantify miRNA in terms of absolute copy numbers. It involves the partitioning of a cDNA sample into multiple parallel PCR reactions. The reaction is performed with nanofluidics partitioning or emulsion chemistry, based on the random distribution of the sample on a specific support. It is superior to previously described methods in terms of sensitivity and precision, and it is the technique most widely used to study miRNA expression in plasma or serum samples, where there are no stable endogenous controls [61].

## 4. miRNAs as Diagnostic Markers in Thyroid Cancer

After clinical and ultrasound assessment of the likelihood of malignancy, most thyroid nodules are subjected to FNAB for cytological examination [62, 63]. This approach has shown good accuracy in discriminating most DTCs from benign lesions. However, in a nonnegligible proportion of cases, the cytology is indeterminate [10]. In these cases, the evaluation of molecular markers in the aspirate can often allow more confident presurgical differentiation of benign and malignant lesions. miRNAs are one of the novel classes of molecular markers that are being used to improve the diagnosis of thyroid cancer [13, 64]. Several studies have shown that a miRNA-based signature in FNABs can be used to discriminate benign from malignant thyroid nodules (Table 2).

A recent meta-analysis [71] of 543 patients with benign (*n* = 277) or malignant (*n* = 266) thyroid nodules indicates that miRNA analysis of fine-needle aspirates (FNAs) allows significantly more accurate individuation of the malignant lesions than conventional cytology. More recently, Paskas et al. [70] assessed the performance of a panel of four markers, the BRAF V600E mutation, miRNA-221, miRNA-222, and galectin-3 protein, developing an algorithm for distinguishing benign and malignant thyroid nodules. In particular, among the 120 nodules of the study, the proposed algorithm classified 62 cases as benign (against the 75 cases observed with the conventional cytology classification), 9 false negative cases, and 6 false positive cases, with a sensitivity of 73.5%, a specificity of 89.8%, and an accuracy of 75.7%, thereby allowing over half the patient cohort to avoid unnecessary surgery. In a cohort of 118 samples of PTCs, Panebianco et al. [69] developed a statistical model that accurately differentiates malignant from benign indeterminate lesions on thyroid FNAs using a panel of two miRNAs and two genes (miRNA-146b, miRNA 222, KIT, and TC1). More recently, Stokowy et al. [64] have identified that two miRNA markers might improve the classification of mutation-negative thyroid nodules with indeterminate FNA. In this study, it was observed that miRNA-484 and miRNA-148b-3p identify thyroid malignancy with a sensitivity of 89% and a specificity of 87% in a subset of 44 FNA samples.

As for ATCs, most of the studies conducted thus far have failed to produce statistically significant data since the number of tumor samples examined is invariably low [67, 72]. At present, no data are available on the potential of miRNA assays for diagnosis of MTC.

Recently, improved diagnosis of cancer has been achieved by assaying cancer-derived materials isolated from peripheral blood samples [73]. These “liquid biopsies” provide a genetic snapshot of the whole-tumor landscape, including both primary and metastatic lesions [74]. Relatively few reports are available on the expression and clinical significance of circulating miRNAs in patients with thyroid cancer, particularly those with less common tumors, such as MTC, PDTC, and ATC. As shown in Table 3, the studies published to date have focused mainly on patients with PTC, but the results are nonetheless characterized by high variability. Several elements can contribute to these highly variable results, including the number of patients of each study and/or sample-related factors (i.e., gender, sample collection time), preanalytical factors (i.e., sample type, storage conditions, and/or sample processing), and experiment-related factors (i.e., RNA extraction protocol, quantification methods). In addition, only few studies reported the isoforms of the miRNAs identified. Circulating levels of miRNA-146b-5p, miRNA-221-3p, and miRNA-222-3p in PTC patients have been found to be higher than those in healthy controls [75, 76, 84], while miRNA-222 and miRNA-146b levels also reportedly discriminate between PTCs and benign nodules [75, 80, 83]. Plasma levels of miRNA-21 in FTC patients are reportedly higher than those found in patients with benign nodules or PTC, whereas miRNA-181a is more highly expressed in PTC patients than in those with FTC [81]. In PTC patients, circulating levels of miRNA-146a-5p, miRNA-146b-5p, miRNA-221-3p, and miRNA-222-3p have been shown to decline after tumor excision [75, 76, 83, 84].

## 5. miRNAs as Prognostic Markers in Thyroid Cancer

miRNA profiling of thyroid cancers can also provide prognostic information useful for defining optimal management strategies. Recent studies have demonstrated that expression levels of certain miRNAs in thyroid tumor tissues are associated with clinic-pathological characteristics, such as tumor size, multifocality, capsular invasion, extrathyroidal extension, and both lymph node and distant metastases. Tumor size displays associations with tissue levels of miRNA-221, miRNA-222, miRNA-135b, miRNA-181b, miRNA-146a, and miRNA-146b [35, 85–87]. The latter two are also associated with multifocality [86, 87]. The single study that analyzed the association between miRNA expression and capsular or vascular invasion identified three miRNAs (miRNA-146b, miRNA-221, and miRNA-222), whose levels were significantly elevated in tumor tissue samples of PTC that had invaded vascular structures and/or the capsule. Extrathyroidal extension has been associated with higher levels of miRNA-221, miRNA-222, miRNA-146a, miRNA-146b, miRNA-199b-5p, and miRNA-135b [35, 87–89]. Expression levels of miRNA-221, miRNA-222, miRNA-21-3p, miRNA-146a, miRNA-146b, and miRNA-199b-5p are reportedly higher in patients with lymph node metastases [85, 86, 89, 90], and miRNA-146b and miRNA-221 are also associated with the presence of distant metastases [86]. Chou and coworkers showed that overall survival rates among patients with higher miRNA-146b expression levels are significantly decreased relative to those associated with lower tumor levels of this miRNA. Overexpression of miRNA-146b significantly increases cell proliferation, migration, and invasiveness and causes resistance to chemotherapy-induced apoptosis [91]. Higher levels of miRNA-146a, miRNA-146b, miRNA-221, and miRNA-222 display positive associations with higher TNM stage (III/IV versus I/II) [35, 85–87]. Risk of recurrence, defined according to the American Thyroid Association (ATA) guidelines, has been positively associated with higher expression of miRNA-146b-5p, miRNA-146b-3p, miRNA-21-5p, miRNA-221, miRNA-222-3p, miRNA-31-5p, miRNA-199a-3p/miRNA-199b-3p, miRNA-125b, and miRNA-203 and lower expression levels of miRNA-1179, miRNA-7-2-3p, miRNA-204-5p, miRNA-138, miRNA-30a, and let-7c [37, 92].

Notably, the studies and findings discussed above are related exclusively to PTCs (Table 4).

Only two studies have investigated the role of miRNAs as prognostic markers in FTC and MTC [52, 93]. The study by Jikuzono and coworkers involved a comprehensive quantitative analysis of miRNA expression in tumor tissue from minimally invasive FTCs (MI-FTC) [93]. The subgroup of tumors that had metastasized (*n* = 12) exhibited significantly higher levels of miRNA-221-3p, miRNA-222-3p, miRNA-222-5p, miRNA-10b, and miRNA-92a than the nonmetastatic subgroup (*n* = 22). Expression of these miRNAs was also upregulated in widely invasive FTCs (WI-FTC; *n* = 13), which are characterized by distant metastasis and a worse prognosis. Logistic regression analysis identified one of these miRNAs, miRNA-10b, as a potential tool for predicting outcomes in cases of metastatic MI-FTC [93]. The second study, conducted by Abraham and coworkers, found that overexpression of miRNA-183 and miRNA-375 in MTCs (*n* = 45) was associated with lateral lymph node metastasis, residual disease, distant metastases, and mortality [52].

Recent studies have also looked at circulating miRNAs in patients with PTCs, which are showing undeniable promise as novel predictors of early disease relapse (Table 5).

In these patients, circulating levels of miRNA-146b-5p, miRNA-221-3p, miRNA-222-3p, and miRNA-146a-5p have been shown to decline after tumor excision [75, 76, 81, 83, 84]. Notably, serum levels of miRNA-221-3p and miRNA-146a-5p also appear to predict clinical responses to treatment, with significantly increased levels observed at the 2-year follow-up in PTC patients with structural evidence of disease, including some whose serum thyroglobulin assays remained persistently negative [84]. The association of thyroid cancer with circulating levels of miRNA-146b-5p, miRNA-221-3p, and miRNA-222-3p has been strengthened by evidence of their upregulated expression in PTC [37, 94], FTC [27, 95], and ATC [27] tissues and by their association with tumor aggressiveness.

## 6. Conclusion

Analysis of miRNA expression levels and detection of circulating miRNAs can be used for the early diagnosis of thyroid cancer and for monitoring treatment responses. Compared with circulating messenger RNAs, circulating miRNAs are emerging as more promising biomarker candidates because they are more stable and tissue specific. miRNAs can also be assessed in other biological samples, such as FNABs, which can be obtained with minimally invasive procedures to easily identify a specific profile, which makes specific miRNAs optimal diagnostic and/or prognostic biomarkers. Improved standardization of methods used to assay circulating miRNAs will allow more extensive use of this approach in defining individualized treatment strategies for thyroid cancer patients.

## Figures and Tables

**Table 1:** Known targets for deregulated miRNAs in thyroid tumors and association with genetic alterations.

Histotype	miRNA expression (↑/↓)^∗^	Oncogenic alteration	Molecular target	Reference
PTC	↑ 146b, 221, 222	n. d.	KIT	[24]
	↑ 181b, 221, 222	n. d.	n. d.	[43]
	↑ 187	RET/PTC, RAS	n. d.	[27]
	↑ 146b, 221, 222; ↓ 187	BRAF V600E		
	↑ 146b	BRAF V600E	n. d.	[33]
	↑ 221	BRAF V600E	n. d.	[34]
^∗∗^	↑ 451	n.d.	n.d.	[44]
	↓ 137	n. d.	CXCL12	[45]
	↓ 451a	n.d.	n.d.	[46]
FTC	↑ 197, 346	n. d.	n. d.	[42]
	↑ 181b, 187	n. d.	n. d.	[27]
	↑ 221	n. d.	n. d.	[29]
	↓ 574-3p			
	↑ 146b, 183, 221	n. d.	n. d.	[38]
	↓ 199b			
	↓ 199a-5p	n. d.	CTGF	[47]
Hürtle	↑ 187, 197	n. d.	n. d.	[27]
	↑ 885-5p	n. d.	n. d.	[29]
	↑ 885-5p	n. d.	n. d.	[40]
	↓ 138, 768-3p			
ATC	↑ 137, 205, 302c	n. d.	n. d.	[27]
	↑ 221, 222	n. d.	n. d.	[48]
	↑ 146a	n. d.	NF-kB	[49]
	↓ 30, 200	n. d.	n. d.	[39]
MTC	↑ 130a, 138, 193a-3p, 373, 498	n. d.	n. d.	[50]
	↓7, 10a,29c, 200b-200c			
	↑ 9, 21, 127, 154, 183, 224, 323, 370, 375	n. d.	PDCD4	[28, 51]
	↓ 129-5p	RET	n. d.	[30]
	↑ 183, 375	n. d.	n. d.	[52]
	↑ 10a, 375	n.d.	YAP	[53]
	↓455			

(^∗^) ↑/↓: upregulated/downregulated; (^∗∗^): PTC with lymph node metastasis. ATC: anaplastic thyroid carcinoma; BRAF: b-type rapidly accelerated fibrosarcoma; CTGF: connective tissue growth factor; CXCL12: C-X-C motif chemokine ligand 12; FTC: follicular thyroid carcinoma; KIT: proto-oncogene receptor tyrosine kinase; MTC: medullary thyroid carcinoma; n. d.: not determined; PDCD4: programmed cell death 4; PTC: papillary thyroid carcinoma; RAS: rat sarcoma; RET/PTC: rearranged during transfection/papillary thyroid carcinoma; YAP: yes-associated protein.

**Table 2:** Studies of miRNAs in FNAB samples.

Samples	Histological diagnosis^∗^	miRNA expression (↑/↓)^∗∗^	Reference
8 (malignant)	PTC	↑ 181b, 221, 222	[43]
62 (8 malignant, 5 benign, 49 n.d.)	7 PTC, 1 Hürtle	↑ 146b, 155, 187, 197, 221; 222, 224	[27]
115 (37 malignant, 78 benign)	10 FTC or Hürtle (27 n.d.)	↑ 138	[65]
27 (20 malignant, 7 benign)	PTC	↑ 221	[66]
128 (88 malignant, 40 benign)	3 ATC, 13 FTC, 72 PTC	↑ 146b, 187, 221	[67]
		↓ 30d	
141 (58 malignant, 83 benign)	58 PTC	↑ 146b, 155, 221	[68]
118 (70 malignant, 48 benign)	70 PTC	↑ 146b, 222	[69]
120 (45 malignant, 75 benign)	1 FTC, 2 ATC, 4 MTC, 8 Hürtle, 30 PTC	↑ 221, 222	[70]
44 (24 malignant, 20 benign)	24 FTC	↓ 148b-3p, 484	[64]

(^∗^) related to malign samples; (^∗∗^) ↑/↓: upregulated/downregulated. ATC: anaplastic thyroid carcinoma; FNAB: fine-needle aspiration biopsy; FTC: follicular thyroid carcinoma; MTC: medullary thyroid carcinoma; PTC: papillary thyroid carcinoma.

**Table 3:** Circulating miRNAs as diagnostic biomarkers in thyroid carcinoma.

Histotype	Sample type	miRNA	Up/downregulated	Reference
PTC	Serum	let-7e, 151-5p, 222	Up	[75]
	Plasma	146b, 222	Up	[76]
	Serum	190	Up	[77]
		95	Down	
	Plasma	hsa-let7b-5p, hsa-miR-10a-5p, hsa-miR-93-5p, hsa-miR-191	Up	[78]
		hsa-miR-146a-5p, hsa-miR-150-5p, hsa-miR-199b-3p, has-miR-342-3p	Down	
	Plasma	let-7i, 25-3p, 140-3p, 451a	Up	[79]
	Plasma	146b, 155	Up	[80]
	Plasma-derived exosomes	31-5p, 126-3p, 145-5p, 181a	Up	[81]
	Plasma	9-3p, 124-3p	Up	[82]
	Serum	222	Up	[83]
		21	Down	
	Serum	24-3p, 28-3p, 103a-3p, 146a-5p, 146b-5p, 191-5p, 221-3p, 222-3p	Up	[84]
FTC	Plasma-derived exosomes	21	Up	[81]

FTC: follicular thyroid carcinoma; PTC: papillary thyroid carcinoma.

**Table 4:** Tissue miRNAs as prognostic biomarkers in PTC.

miRNA	Tumor size	Multifocality	Capsular invasion	Vascular invasion	ETE	LN metastases	Distant metastases	Overall survival	TNM stage	ATA risk	References
1179										^∗^	Rosignolo et al. [37]
125b										^∗^	Geraldo and Kimura [92]
135b	^∗^				^∗^						Wang et al. [35]
138										^∗^	Geraldo and Kimura [92]
146a		^∗^			^∗^	^∗^			^∗^		Sun et al. [87]
146b	^∗^	^∗^	^∗^	^∗^	^∗^	^∗^	^∗^	^∗^	^∗^	^∗^	Wang et al. [35], Acibucu et al. [86], Sun et al. [87], Chou et al. [91], Geraldo and Kimura [92]
146b-3p										^∗^	Rosignolo et al. [37]
146b-5p										^∗^	Rosignolo et al. [37]
181b	^∗^										Sun et al. [85]
199a-3p/199b-3p										^∗^	Rosignolo et al. [37]
											Rosignolo et al. [37]
199b-5p					^∗^	^∗^					Peng et al. [89]
203										^∗^	Geraldo and Kimura [92]
204-5p										^∗^	Rosignolo et al. [37]
21										^∗^	Geraldo and Kimura [92]
21-3p						^∗^					Huang et al. [90]
21-5p										^∗^	Rosignolo et al. [37]
221	^∗^		^∗^	^∗^^∗^	^∗^	^∗^	^∗^		^∗^	^∗^	Wang et al. [35], Sun et al. [85], Acibucu et al. [86], Wang et al. [88], Geraldo and Kimura [92]
222	^∗^		^∗^	^∗^	^∗^	^∗^			^∗^	^∗^	Wang et al. [35], Sun et al. [85], Acibucu et al. [86], Wang et al. [88], Geraldo and Kimura [92]
222-3p										^∗^	Rosignolo et al. [37]
30a										^∗^	Geraldo and Kimura [92]
31-5p										^∗^	Rosignolo et al. [37]
7–2-3p										^∗^	Rosignolo et al. [37]
let-7c										^∗^	Geraldo and Kimura [92]

^∗^: information included in indicated studies; ATA: American Thyroid Association; ETE: extrathyroidal extension; LN: lymph node.

**Table 5:** Circulating miRNAs as prognostic biomarkers for PTC follow-up.

Study	Number of cases	Samples	miRNA	Findings
Yu et al. [75]	9	Pre- and postoperative serum (5–15 days)	151-5p, 222	Decreased after tumor excision
Lee et al. [76]	32	Pre- and postoperative plasma (2–6 weeks)	221, 222, 146b	Decreased after tumor excision
Li et al. [79]	7	Pre- and postoperative plasma (4–7 days)	25-3p, 451a	Decreased after tumor excision
Samsonov et al. [81]	10	Pre- and postoperative plasma-derived exosomes (7–10 days)	126-3p, 145-5p, 146a-5p, 181a-5p, 206, 21-5p, 221-3p, 223-3p, 31-5p	Decreased after tumor excision
Yoruker et al. [83]	31	Pre- and postoperative serum (5 weeks)	221, 222, 151-5p, 31	Decreased after tumor excision
Rosignolo et al. [84]	44	Pre- and postoperative serum (30 days)	146a-5p, 221-3p, 222-3p, 146b-5p, 28-3p, 103a-3p, 191-5p, 24-3p	Decreased after tumor excision

PTC: papillary thyroid carcinoma.
